# 

**Published:** 1903-03

**Authors:** 


*THE BRISTOL MEDICO-CHIRURGICAL SOCIETY.*

### CONTENTS.

| | PAGE |
|---|---|
| **Therapeutics of a Sea Voyage.** By R. ROXBURGH, M.B. Ed., F.R.C.S. Ed. ... | 1 |
| **On the Probable Rhythmical Contraction of the Bronchial Muscular Coat as a Factor in Pulmonary Disease.** By P. WATSON WILLIAMS, M.D. Lond. ... | 6 |
| **A Case of Banti's Disease, with Autopsy.** By J. MICHELL CLARKE, M.A., M.D. Cantab., F.R.C.P. ... ... ... ... ... | 14 |
| **Remarks on the "Light Treatment" of Lupus, Vulgaris and Erythematosus, and Rodent Ulcer, &c.** By A. J. HARRISON, M.B. Lond. ... ... ... ... | 23 |
| **Perforated Typhoid Ulcer.** By H. G. KYLE, M.B., B.Ch. Oxon.... ... ... ... | 30 |
| **On Some of the Initial Signs of Pleurisy.** By F. H. EDGEWORTH, M.B., B.A. Cantab., B.Sc. Lond. ... ... ... ... | 36 |
| **Two Cases of Ruptured Tubal Pregnancy.** By HAROLD F. MOLE, F.R.C.S. | 39 |
| **Progress of the Medical Sciences:** | |
| **Medicine.** THEODORE FISHER, M.D. Lond. ... ... ... ... ... ... ... ... ... | 44 |
| **Surgery.** J. LACY FIRTH, M.D., M.S. Lond., F.R.C.S. ... ... ... ... ... | 48 |
| **Reviews of Books** ... ... ... ... ... ... | 54 |
| **Editorial Notes** ... ... ... ... ... ... | 71 |
| **Notes on Preparations for the Sick** ... | 79 |
| **The Library of the Bristol Medico-Chirurgical Society** ... .. ... ... | 81 |
| **Meetings of Societies** ... ... ... ... ... | 83 |
| **Obituary** ... ... ... ... ... ... ... ... ... | 94 |
| **Local Medical Notes** ... ... ... ... ... | 95 |

